# The Great War: Wonderful Progress in Evacuation and Care of the Wounded

**Published:** 1918-02-09

**Authors:** 


### THE GREAT WAR.

### Wonderful Progress in Evacuation and Care of the Wounded.

The wounded are now more expeditiously and comfortably evacuated from the battlefield and more carefully attended to at the consecutive receiving posts than at any previous time in the history of war. During a recent visit to the British sphere of operations in France we were much struck by the changes in the character of the various medical establishments all along the line of evacuation from Front to Base, and by their gradual development from forwarding posts and agencies of the rough-and-ready type to miniature hospitals modelled on the highest metropolitan standards. The medical transport has also evolved on similar lines; motor ambulance wagons have improved in type and fittings, and those in the field are heated by pipes connected with the exhaust from the engine. The network of light railways is also utilised, and miniature ambulance trains with closed and heated wagons run up to the front area, in some instances to within 600 to 800 yards of the firing line, and transport the wounded direct to the casualty clearing stations.

### A Typical Regimental Aid Post.

Battalions are responsible for collecting their wounded in the first instance. The battalion stretcher-bearers, after applying first dressings, convey them on stretchers to the regimental aid post. This is the headquarters of the battalion medical officer, situated on the line of communication trenches as near the headquarters of the battalion commander as possible, and from 600 to 1,000 yards from the firing line; it is located in a dug-out of any depth up to 40 feet, or in exceptional cases in the cellars of demolished buildings, and has accommodation for operating and dressing tables, for stretcher cases on racks, and for the medical officer and his staff of bearers. A typical one we visited in the Somme area was in the form of a gallery, 4 feet wide by 6 feet high, with separate entrance and exit, and with the various compartments excavated on either side, the whole of the structure being revetted with wooden props and beams. The surgical room was lined with green rotproof canvas, lighted by acetylene gas and heated by oil stoves. It is not always, however, that this luxurious type is possible. The labour and time involved in the construction of a model regimental aid post are very considerable, and frequently before it is finished there is a change in the line and all has to be recommenced in another place. Then the constant change of troops is a source of interruption; men do not work so hard when they do not anticipate the enjoyment of the results of their labours. In the case of an advance the regimental aid post is established in the conquered German trenches, the dug-outs in which are even more elaborate than our own; some of those taken over on the Somme were veritable underground mansions of two or three storeys, dry, warm, and lit by electricity.

### What Scientists have Accomplished.

The devoted and continued labours of the scientists who have been engaged on research into the causes of surgical shock are bearing fruit, to the infinite advantage of our wounded heroes. The means of combating it which are practicable are rest, warmth, sedatives, and the fixation of injuries, and attempts are being made to apply these at the earliest possible moment—even from regimental aid posts. Shock cases are provided with hot-water bottles and blankets, and when the military situation permits they are detained in a compartment warmed by oil stoves until reaction occurs, when they are given hot tea and morphia, and dispatched, well wrapped up in blankets and waterproof sheeting, to the advanced dressing station, where, we shall learn anon, further steps are taken before they are put into ambulance cars or trains.

### The Fixation of Injured Limbs.

The fixation of injuries of the limbs complicated by fractures is secured by Thomas' splints, complete outfits of which, including suspension bars and heel extension hooks, are included in numbers sufficient to meet all emergencies in the equipment of every medical establishment, from the regimental aid post to the base; the universal adoption of this method of preparing wounded limbs for transport has done much to mitigate the suffering of the wounded soldier; the contrast in the feelings of two men suffering from similar injuries, travelling in an ambulance wagon, the one wearing the old long Liston splint and the other the Thomas, is indescribable. Serious injuries uncomplicated by fractures are supported by Gooch splinting. These anti-shock measures have resulted in the delivery of the wounded at casualty clearing stations in a better condition for operation than ever before, and the deaths attributable to surgical shock have already been reduced by 25 per cent.

During ordinary trench warfare the wounded are taken back from the regimental aid post to the advanced dressing station by R.A.M.C. bearers, a party of whom are permanently stationed there for this purpose. During active operations R.A.M.C. bearer divisions assist the regimental bearers in clearing the battlefield. The means of conveyance are hand stretchers, wheeled stretcher carriers, motor ambulance wagons, horsed ambulance wagons, or the trench railway system, according to circumstances. The usual distance of the advanced dressing station from the regimental aid post is about 3,000 yards, and when hand carriage is employed relays of bearers are stationed every 600 to 800 yards along the communication trenches.

### Developments in Advanced Dressing Stations.

The advanced dressing station is gradually developing into a miniature hospital. It is usually located in extensive dug-outs or cellars with compartments for a surgical dressing-room, detention

**THE GREAT WAR**—(*continued*).

racks for thirty to forty stretcher cases, and accommodation for two or more medical officers and from forty to sixty bearers. It is warmed and well lit by acetylene gas and provided with hot-air apparatus for the resuscitation of shock cases, and a large supply of hot-water bottles or heated bricks. Here every case is investigated, anti-tetanic serum is given, particulars entered on a card, and those requiring attention prepared for the journey to the casualty clearing station. As an instance of what is possible in this way, we were informed that during the recent active operations, at one A.D.S. most of the stretcher cases, 300 in number, had their wet clothing removed and were dressed in pyjamas for the journey, and that at another A.D.S. ninety were similarly dealt with.

### Destinations of the Wounded.

The destination of wounded evacuated from advanced dressing stations differs in the various army corps, and even divisions: until quite recently it was the invariable rule to transmit them to "main dressing stations," as the divisional field ambulances which are open for the reception of wounded are designated, which involved further disturbance before their arrival at the C.C.S., where they can be properly dealt with. Main dressing stations are located from 6,000 to 8,000 yards from the advanced dressing stations, in schools or similar buildings, or in huts. They nominally accommodate 150 patients on palliasses, but it is quite common for them to expand their accommodation to 400-500 and their standard of equipment to that of a casualty clearing station, including spring beds, hair mattresses, feather pillows, white sheets, dining-halls, and variety theatres. On these lines they subserve a very useful purpose in preventing the loss to the divisions of light cases of sickness which are likely to recover in a fortnight; but the casualty clearing stations are now pushed much farther up than formerly, and are in some cases in front of the divisional main dressing stations, and the tendency is to leap-frog the dressing stations, in the case of wounded, and evacuate direct from the advanced dressing station to the casualty clearing stations, the advantages of which are so obvious that it is difficult to imagine why this course was not adopted before.

### From Collecting Depôts to Clearing Stations.

Casualty clearing stations have gradually evolved since 1914 from mere collecting depôts for the wounded awaiting transport to the lines of communication by ambulance trains to highly specialised institutions where each case is considered from the aspect of the comfort and well-being of the individual, and the immediate surgical measures necessary to place him on the shortest road to recovery. The patients are received in spacious and well-heated huts, hangars, or cunningly joined marquees, some of them capable of accommodating as many as 1,000 at a time. Here their particulars are noted, and injuries sorted, in accordance with the action required. For instance, abdominal cases take precedence; if they are fit for operation they are taken at once to the preparation-room, and from there to the operating-room. If there should be symptoms of shock they are first directed to the special department which deals with this condition. Here there are bed cradles fitted with electric bulbs which are connected in pairs with the current in order to ensure control over the temperature; these are capable of promoting complete reaction ordinarily in fifteen to twenty minutes. "Gassed" cases are dealt with in a special department provided with Haldane's oxygen apparatus.

### Organisation and Methods of Work.

During active operations the casualty clearing station presents a very lively scene. Surgeons, nurses, and bearers are called in from field ambulances located in parts of the line which are not heavily engaged. Operating teams, each consisting of an operating surgeon, assistant-surgeon, anæsthetist, nursing sister, and trained R.A.M.C. operating-room attendant, expand from one to as many in some instances as twenty. The teams work during the height of the battle in sixteen-hour shifts, and for the few days during its continuance they have a trying and strenuous time, but in the frequently long intervals between serious battles there is an equally trying enforced period of rest. Casualty clearing stations, when there are large numbers of wounded pouring in, work in groups; 100, 200, 300 wounded are admitted to each C.C.S. in rotation. This distribution of work ensures all the rest to the staff that is possible under the circumstances; it also enables the consulting surgeon to attend each C.C.S. and give advice in difficult cases, an incalculable benefit both to the patient and surgeon; it is Harley Street in the field. The operating rooms and equipment are models of surgical smartness and comfort. The lighting by day and night is very good, and many of the operating-tables are heated by electricity. An *x*-ray installation is attached to each C.C.S., and in some an illuminated plate of the injuries is displayed in the operating-room during the operation.

### Surgical Wards, and the Feeding Arrangements, etc.

The surgical wards are up to the standard of the best Metropolitan hospitals. Enamelled-iron spring beds, with air-mattresses, pillows, sheets and artistic covers, and tables and arm-chairs are provided by the Red Cross Society, and the nursing sisters generally manage, to procure flowers and other decorations. The feeding arrangements are wonderfully good. A full ration is available for those able to eat it, and there are chicken, fresh eggs, fresh milk, and all the usual invalid foods for those requiring them. The convalescents take their meals in a dining-hall accommodating several hundreds. Amusement and recreation are provided by football, cricket, golf, and a new game, played with hand-balls and nets, which is particularly suited for convalescents. Every C.C.S. has a reading-room and

THE GREAT WAR—(*continued*).

a theatre, many have their own band, and most their own variety troupe.

Other Interesting Factors of Importance.

From this description it is difficult to realise that a casualty clearing station is a mobile field unit, but such is the case, and it does advance with the Army, albeit slowly, and for this reason a travelling operating unit, invented by Captain Cowell, R.A.M.C., has been attached to each C.C.S. The equipment, which is very complete, is conveniently packed in cabinets which are carried on a specially fitted trailer attached to a motor lorry which accommodates the operating team—one operating surgeon, one assistant-surgeon, one nursing sister, and one operating-room attendant. This unit is always available to start at any moment, and within a very short time can commence work as an advanced C.C.S., probably provisionally located in a field ambulance dressing station. There are other spheres of usefulness not immediately connected with the wounded which occupy the C.C.S., such as the dental, bacteriological, and eye departments, the medical wards and the medical school, which complete the tribute to progress which is the object of this little sketch.

